# Analysis of Physicochemical Characteristics, Flavor, and Microbial Community of Sichuan Industrial Paocai Fermented by Traditional Technology

**DOI:** 10.3390/foods14183232

**Published:** 2025-09-17

**Authors:** Shuang Xian, Hongchen Li, Xinyi Wang, Xiangchao He, Yanlan Li, Xinyan Liu, Guanghui Shen, Anjun Chen

**Affiliations:** 1 College of Food Science, Sichuan Agricultural University, Ya’an 625014, China; xianshuang@stu.sicau.edu.cn (S.X.); lxy05@126.com (X.L.); shenghuishen@163.com (G.S.); 2 Key Laboratory of Agricultural Product Processing, Nutrition Health (Co-Construction by Ministry and Province), Ministry of Agriculture and Rural Affairs, Ya’an 625014, China

**Keywords:** Paocai, fermentation, fermented vegetables, volatile flavor compounds, regional characteristics

## Abstract

Sichuan Paocai is a representative traditional fermented vegetable in China, which is deeply embedded in local geographical and cultural heritage. However, regional differences in product characteristics remain poorly understood. In this study, the physicochemical properties, volatile compounds, and microbial communities of Paocai from seven production regions in Sichuan (named FB, AB, BZ, CD, DZ, MY, and YS) were systematically investigated. Parameters including pH, salinity, nitrite, organic acids, and color were determined, while volatile profiles were analyzed using an electronic nose and comprehensive two-dimensional gas chromatography–mass spectrometry. A total of 294 volatile compounds were identified, with alcohols, esters, and isothiocyanates emerging as the major contributors to flavor differentiation. UMAP and OPLS-DA analyses revealed distinct regional clustering, which was consistent with electronic nose profiling, and 111 volatile compounds were identified as key aroma markers. Microbial diversity was assessed using 16S rRNA gene sequencing, demonstrating that *Lactobacillus*, *Lentilactobacillus*, *Pediococcus*, and *Weissella* were the dominant taxa, although the richness varied significantly across regions. An LEfSe analysis further identified region-specific biomarkers, including *Pediococcus*, *Lactococcus*, and *Leuconostoc* in FB; *Lactobacillus* in AB; *Pediococcus ethanolidurans* in BZ; *Levilactobacillus* in DZ; *Lentilactobacillus* in MY; and a more diverse microbiota in MS. A correlation analysis highlighted the pivotal roles of distinct microbial groups in shaping and transforming flavor compounds across different regions. Overall, these findings provide scientific guidance for the development of high-quality, region-specific products and contribute to the protection, branding, and market competitiveness of geographically indicated foods.

## 1. Introduction

Traditional Paocai is a hallmark of Chinese fermented vegetables, produced primarily from fresh vegetables using time-honored techniques, such as spontaneous fermentation, inoculation aged brine as a starter, fermentation in pottery jars, and natural temperature regulation [1]. Among these, Sichuan Paocai (CSP) is particularly renowned in China for its diverse raw materials, distinctive aroma, crisp texture, and straightforward production process. With the advancement of industrial techniques, CSP has reached large-scale production, accounting for nearly 70% of the national Paocai market [2], and has become a cornerstone of Sichuan cuisine and a culturally significant food heritage.

To satisfy the demands of industrial-scale and standardized production, modern CSP manufacturing frequently employs high-salt processing: vegetables are initially marinated in 10–15% brine to yield semi-finished products, followed by desalination (to 5–8% salt), seasoning, sterilization, and packaging [3,4]. While these measures safeguard the food safety and supply chain stability, they also introduce critical challenges. First, the release of high-salinity wastewater contributes to environmental pollution. Second, flavor profiles tend to homogenize, losing the multifaceted sensory characteristics that are unique to traditional fermentation. Recent studies report that industrial Paocai exhibits markedly reduced microbial diversity [5,6], accompanied by lower concentrations and diversity of volatile and non-volatile metabolites compared with homemade products [5,7]. The most distinctive advantage of the traditional process—the regulatory function of aged brine (back-slopping starter)—is increasingly recognized as a potential solution to these issues [7]. Mature aged brine, obtained through repeated vegetable additions and salt replenishment, develops a stable microbial community after approximately four fermentation cycles [1,8], dominated by lactic acid bacteria and displaying enhanced metabolic activity relative to fresh brine [6]. Unlike kefir grains or sourdough [9,10], aged brine lacks a fixed physical matrix and remains separate from the final product, facilitating its integration into industrial workflows. Although the stability of spontaneous fermentation products has long been questioned, the incorporation of traditional fermentation strategies into industrial production is now viewed as a promising pathway for upgrading the sector. Yet, whether the unique sensory profile of traditional household Paocai can be reproduced under large-scale conditions remains an unresolved scientific challenge.

In spontaneous fermentation systems, the assembly and succession of microbial communities are jointly shaped by terroir, raw materials, and process parameters [11]. Terroir influences not only the phenotypic characteristics of the raw ingredients but also the regulation of fermentation process parameters [11]. While widely recognized as the foundation for regional identity and geographical indication (GI) protection, the direct mechanistic contribution of terroir to product quality has been insufficiently explored. Sichuan Paocai was awarded “China Geographical Indication Product” status in 2010 and was listed under the China–EU GI Agreement in 2020. Nevertheless, Sichuan encompasses diverse ecological subregions, such as the Chengdu Plain, South Sichuan, and North Sichuan, with substantial variation in climate, hydrology, soil composition, and environmental microbial reservoirs. These factors can directly modulate the colonization dynamics and successional trajectories of fermentative microbiota [12,13], or indirectly alter vegetable physicochemical properties (e.g., pectin and cellulose content) [14], thereby influencing substrate bioavailability and microbial metabolic performance. However, a systematic comparison of how terroir—the distinct environmental conditions of different Sichuan subregions—affects the quality of industrially produced Paocai, particularly with respect to variations in physicochemical properties, flavor compound profiles, and core microbial communities, while contrasting with home-scale spontaneously fermented counterparts, is still lacking.

To address this gap, the objectives of this study were to: (1) systematically compare the physicochemical properties, volatile compound profiles, and microbial community structures of industrial Paocai from different subregions of Sichuan; (2) identify the key volatile metabolites and microbial biomarkers contributing to regional differentiation; and (3) elucidate the correlations between dominant microbial taxa and flavor-active compounds to elucidate the mechanisms by which regional environmental variation influences Paocai quality under industrial conditions. The findings will provide a theoretical basis for optimizing fermentation processes while preserving traditional flavor complexity, and offer scientific guidance for developing high-quality, region-specific products, thereby supporting the protection, branding, and market competitiveness of geographical indication foods.

## 2. Materials and Methods

### 2.1. Paocai Preparation and Sampling

From June to July 2025, industrially produced and spontaneously fermented Paocai were collected from five renowned food companies specializing in large-scale Sichuan Paocai production, located in Bazhong City (BZ), Chengdu (CD), Dazhou City (DZ), Mianyang City (MY), and Meishan City (MS), Sichuan, China. All the companies employed traditional production methods: washed, drained, and cut raw vegetables and spices (such as red pepper, garlic, ginger, and Chinese pepper) were placed into pottery jars containing 4–8% (*w*/*v*) brine. Each pottery jar typically accommodated no less than 50 kg of product. Subsequently, a water seal was applied to the jar rim, allowing the fermentation to proceed at room temperature. Home-scale aged brine fermented Paocai (AB) and fresh brine fermented Paocai (FB) were prepared as controls, following our previously described method [1]. For industrial samples, at least six pre-packaged bags from the same production batch (from multiple independent fermentation jars) were randomly selected; every two bags were combined to form one composite sample, yielding three technical replicates. Radish Paocai solids and brine were processed separately. All the samples were sealed in sterile plastic bags and immediately transported to the laboratory on dry ice. Control samples were obtained from three independent fermenters, with three biological replicates for each control type (AB and FB). For the physicochemical and flavor analyses, radish Paocai solids were homogenized, while brine samples were centrifuged at 12,000× *g* for 10 min at 4 °C to separate the supernatant and cell pellet. The supernatant was used for pH measurement, and the cell pellet was used for RNA extraction. All the processed samples were immediately stored at −80 °C until further analysis.

### 2.2. Determination of Physiochemical Properties and Non-Volatile Compounds

Titratable acidity (TA) and nitrite content were measured in accordance with the Chinese national standards of GB 12456–2021 [15] and GB 5009.33–2016 [16], respectively. The pH, salinity, and color parameters were determined using a pH meter (PHS-3C, INESA Scientific Instrument, Shanghai, China), a multi-parameter analyzer (DZS-706, INESA Scientific Instrument, China), and a colorimeter (NR60CP, Threenh Technology, Shenzhen, China), respectively. Organic acids were quantified following the procedure described by Xian, et al. [17].

### 2.3. Determination of Volatile Organic Compounds

An electronic nose (E-nose; CNose, Shanghai Baosheng Industrial Development Co., Ltd., Shanghai, China) was used to profile volatile aroma compounds. The instrument was equipped with 18 metal oxide semiconductor (MOS) sensors; the sensor characteristics are summarized in Table 1. The sample preparation and analytical parameters were as follows: a 1.00 g aliquot of homogenized Paocai was weighed into a 15 mL headspace vial, vortexed for 60 s, equilibrated in a water bath at 30 °C for 30 min, and subsequently analyzed using the E-nose. The carrier gas (zero air) flow rate was set to 1.0 L·min^−1^; the data acquisition time was 120 s; and the sensor cleaning time was 120 s. During cleaning, it was essential to ensure that the response value of each sensor was normalized to 1. Each sample was analyzed in six technical replicates (*n* = 6).

Volatile organic compounds (VOCs) were analyzed using headspace solid-phase microextraction coupled with comprehensive two-dimensional gas chromatography mass spectrometry (HS-SPME/GC–MS) using a GCMS-QP2020 NX system (GC-2030, Shimadzu, Shanghai, China). Sample pretreatment was performed with minor modifications to the method described in Xian, et al. [18]. Briefly, a 5.00 g aliquot of homogenized Paocai was weighed into a 15 mL headspace sampling vial, 1.00 g of sodium chloride was added to increase the ionic strength, and 100 μL of cyclohexanone (0.554 ug/μL; internal standard) were introduced. The vial was vortexed for 1.0 min and equilibrated in a constant temperature water bath at 50.0 °C for 10.0 min. After that, a 1 cm 50/30 um DVB/CAR/PDMS SPME fiber was exposed to the headspace and used to extract VOCs for 30 min at 50.0 °C. The fiber was then withdrawn and thermally desorbed in the GC injector at 260 °C for 6.0 min in splitless mode. High-purity helium (>99.999%) was used as the carrier gas. The GC operating conditions were: pressure 81.5 kPa; linear velocity 35.7 cm/sec; purge flow 3.0 mL/min. The GC oven was held at 40.0 °C for 3.0 min, then ramped to 240.0 °C at 5.0 °C/min and held for 2.0 min. The mass spectrometry settings were: ion source temperature 230.0 °C; interface temperature 230.0 °C; solvent delay 6.0 min; MS acquisition from 6.0 to 45.0 min in full-scan mode (*m*/*z* 35–350) with a scan interval of 0.03 s (scan speed 20,000).

Retention indices (RIs) were calculated using a C8–C40 n-alkane standard mixture in n-hexane (Solarbio, Beijing Solarbio Science & Technology Co., Ltd., Beijing, China). Compound identification was based on a comparison of the mass spectra and RIs with the NIST library (NIST 2023). Compounds were tentatively identified when the RI difference from the NIST-RI was ≤30 and they met one of the following match-factor criteria: (i) both forward and reverse match factors ≥ 700; or (ii) either the forward or reverse match factor ≥ 800.

### 2.4. DNA Extraction and PCR Amplification

The genomic DNA of microbial communities was extracted from Paocai brine using the E.Z.N.A.^®^ Soil DNA Kit (Omega Bio-tek, Norcross, GA, USA) following the manufacturer’s protocol. DNA quality and concentration were assessed using 1.0% agarose gel electrophoresis and a NanoDrop 2000 spectrophotometer (Thermo Scientific, Waltham, MA, USA), and samples were stored at −80 °C until further analysis. The hypervariable V3–V4 region of the bacterial 16S rRNA gene was amplified using the primer pair 338F (ACTCCTACGGGAGGCAGCAG) and 806R (GGACTACHVGGGTWTCTAAT) with an ABI GeneAmp^®^ 9700 PCR thermocycler (Applied Biosystems, Foster City, CA, USA) [17]. Each 20 µL PCR reaction contained 10 µL of 2× Pro Taq Mix, 0.8 µL of 5 mM forward primer, 0.8 µL of 5 µM reverse primer, 10 ng of template DNA, and nuclease-free water to volume. PCR amplification was performed under the following conditions: initial denaturation at 95 °C for 3 min; 27 cycles of 95 °C for 30 s, 55 °C for 30 s, and 72 °C for 45 s; followed by a final extension at 72 °C for 10 min, and a hold at 4 °C. All the reactions were carried out in triplicate. PCR products were resolved on a 2% agarose gel, purified using a PCR Clean-Up Kit (YuHua, Shanghai, China) according to the manufacturer’s protocol, and quantified using a Qubit 4.0 fluorometer (Thermo Fisher Scientific, Waltham, MA, USA).

### 2.5. DNA Library Construction and Sequencing

Purified PCR products were pooled in equimolar concentrations, and DNA libraries were constructed using the NEXTFLEX Rapid DNA-Seq Kit (Bioo Scientific, Austin, TX, USA) following the manufacturer’s protocol. Sequencing was performed on an Illumina MiSeq PE300 platform (Illumina, San Diego, CA, USA) according to the standard protocols of Majorbio Bio-Pharm Technology Co., Ltd. (Shanghai, China).

### 2.6. Amplicon Sequence Processing and Analysis

Sequencing reads were processed using multiple bioinformatics tools. Low-quality reads were filtered using Fastp (v0.19.6), and paired-end reads were merged using FLASH (v1.2.11). High-quality sequences were denoised using the DADA2 (2024) plugin in the QIIME2 (2024) pipeline with recommended parameters, generating amplicon sequence variants (ASVs) at single-nucleotide resolution based on within-sample error profiles. To minimize sequencing-depth bias on alpha and beta diversity analyses, sequences were rarefied to 6, 8000 per sample, yielding an average Good’s coverage of 99.99%. The taxonomic assignment of ASVs was conducted using the Naive Bayes consensus classifier implemented in QIIME2 against the SILVA 16S rRNA database (v138.2). Based on ASV profiles, rarefaction curves and alpha diversity indices, including observed ASVs, Chao1 richness, the Shannon index, and Good’s coverage, were calculated using Mothur (v1.30.1). Group differences in alpha diversity indices were evaluated using the boot (v1.3-18) and stats (v3.3.1) packages in R (v3.3.1). Beta diversity distance matrices were generated using QIIME2 (v2020.2.0), and a non-metric multidimensional scaling (NMDS) analysis with visualization was performed using the vegan package (v2.4.3) in R. Group differences in beta diversity were further tested using distance matrices derived from QIIME (v1.9.1). The linear discriminant analysis effect size (LEfSe; http://huttenhower.sph.harvard.edu/LEfSe, accessed on 10 August 2025.) was applied to identify the taxa (genera and species) that were differentially enriched among groups (LDA score > 3.5, *p* < 0.05).

### 2.7. Data Processing and Statistical Analysis

Physicochemical parameters and flavor compounds were measured in triplicate, expressed as mean ± standard deviation, and statistically compared using analysis of variance (ANOVA) with Tukey’s multiple comparisons in SPSS Statistics 22 (IBM, Armonk, NY, USA). Graphical presentations were generated using the Origin (v2025; Origin Lab Corporation, Hampton, MA, USA) and R packages, including Venn Package (v1.12), UMAP (v0.2.10.0), ggplot2 (v3.5.1), and MetaboAnalyst (v1.0.1).

## 3. Results

### 3.1. Physiochemical Characteristics Comparison of Spontaneously Fermented Industrial Sichuan Paocai from Different Producing Areas

**Figure 1:**
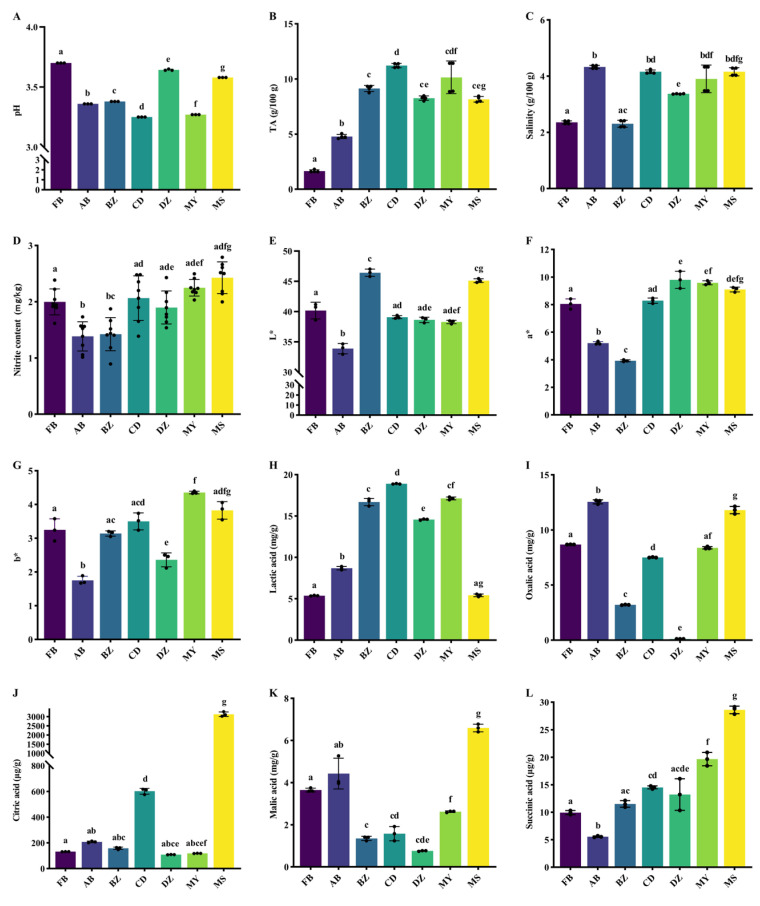
Physicochemical and organic acids at the end of fermentation in spontaneously fermented industrial Sichuan Paocai from different producing areas ((**A**): pH; (**B**): titratable acidity (TA); (**C**): salinity; (**D**): nitrite; and color profile, including (**E**) (lightness (L*)), (**F**) (a* (redness ± greenness)), (**G**) (b* (yellowness ± blueness)); (**H**): Compositions of lactic acid in samples; (**I**): Compositions of oxalic acid in samples; (**J**): Compositions of citric acid in samples; (**K**): Compositions of malic acid in samples; (**L**): Compositions of succinic acid in samples. Different letters indicate the statistical significance at *p* < 0.05. FB represented Paocai fermented by fresh brine; AB represented Paocai fermented by aged brine; BZ, CD, DZ, MY, and MS represented industrial Paocai produced using traditional processes in Bazhong City, Chengdu area, Dazhou City, Mianyang City, and Meishan City, respectively.

Figure 1A,B present the pH and TA of industrially produced Sichuan Paocai from different regions. Overall, pH differed significantly among samples (*p* < 0.05). FB showed the highest pH (~3.70) and the lowest TA (1.6499 g/100 g), and both were significantly different from all other groups, thereby suggesting the lowest degree of acidification. Conversely, CD showed the lowest pH (~3.25) and the highest TA (11.2202 g/100 g), consistent with greater acid accumulation during fermentation. The pH values of AB and BZ were comparable, whereas DZ and MS were significantly higher than CD and MY (*p* < 0.05). The TA values for BZ, DZ, MY, and MS were clustered at ~8–10 g/100 g with no significant differences among them, but all were significantly higher than FB and AB (4.7835 g/100 g) (*p* < 0.05). These differences likely reflect the variation in fermentation duration, microbial community structure, and processing conditions. Salinity is an important determinant of product quality and flavor [19]. As shown in Figure 1C, salinity across regions ranged from 2–5 g/100 g, aligning with previous large-scale surveys of household Paocai [20]. Nitrite is a key safety concern in fermented vegetables. According to the National Food Safety Standard—Limits for Contaminants in Foods (GB 2762–2022 [21]), nitrite (as NaNO_2_) should be <20 mg/kg. In this study, the nitrite levels in all samples were <2.5 mg/kg, with the lowest in AB (1.3838 mg/kg), indicating effective control of nitrite accumulation under industrial conditions.

Color, a critical sensory attribute influencing consumer choice, was characterized by L*, a*, and b* values (Figure 1E–G). L* denotes lightness (higher values indicate brighter appearance); a* indexes the green–red axis (a* > 0 indicates red); and b* indexes the blue–yellow axis (b* > 0 indicates yellow) [22]. The color of fermented products is not only affected by the initial color of the raw materials, but also depends on the changes in pigment during the fermentation process. According to the manufacturer information, all five regions used a red-skinned cultivar of *Red Raphanus sativus* with white flesh. During fermentation, epidermal pigments diffuse inward, yielding a uniform, bright-red appearance that enhances the visual appeal. Among the color parameters, AB showed the lowest L* (lightness), which was markedly lower than the other samples. CD, DZ, MY, and MS demonstrated similar L* and a* values, whereas b* differed significantly among them (*p* < 0.05).

### 3.2. Taste Comparison of Spontaneously Fermented Industrial Sichuan Paocai from Different Producing Areas

Acid production from fermentable sugars by fermentative microbiota is central to vegetable fermentation. Organic acids both stabilize the fermentation environment and shape the product taste [23]. We quantified six key organic acids (tartaric acid, lactic acid, oxalic acid, citric acid, malic acid, and succinic acid) in spontaneously fermented, industrially produced Sichuan Paocai from different regions (Figure 1H–L). Tartaric acid was below the lower limit of the standard curve (30 mg/L) in all the samples. Lactic acid, the principal metabolite of lactic acid bacteria formed via the Embden–Meyerhof–Parnas (EMP) and phosphoketolase (PK) pathways [24], ranged from 5.374 to 18.89 mg/g. CD contained the highest lactic acid (18.89 mg/g), whereas FB contained the lowest (5.374 mg/g). Notably, MS (aged brine fermentation) and FB (fresh brine fermentation) exhibited comparable lactic acid levels (MS ≈ 5.417 mg/g; FB 5.374 mg/g), implying that the fermentation duration may be a major determinant of lactic acid accumulation. Moreover, lactic acid present in the brine likely modulates the acid level in the vegetable matrix: high early-stage acid concentrations can diffuse into tissues, enabling higher accumulation over the same fermentation time, which may explain the elevated lactic acid in BZ, CD, DZ, and MY (~16 mg/g). Oxalic acid, reported to be the predominant organic acid in radish (often >97% of total organic acids) [25], varied widely, from 0.1113 mg/g (DZ) to 12.5463 mg/g (AB). Citric, malic, and succinic acids—core tricarboxylic-acid (TCA) cycle intermediates—were highest in MS, indicating more active organic acid metabolism in that system. Citric acid in MS reached 3129 μg/g, far exceeding other samples (typically <200 μg/g). BZ and DZ demonstrated the lowest malic acid (<1.5 mg/g), suggesting rapid consumption or conversion. Succinic acid was <30 μg/g in all the samples.

To assess the actual contribution of different organic acids to sour perception, taste activity values (TAVs; concentration/threshold) were calculated using the sourness thresholds for each acid (Table 2) [26]. Compounds with TAV > 1 are generally considered to make a perceptible contribution to the overall sourness. Across regions, lactic acids and malic acid had TAVs > 1 and therefore represent the principal contributors to Paocai sourness. In DZ, the TAV of oxalic acid was well below 1, potentially contributing to the observed taste differences. Citric acid imparts a bright, soft sourness [27]; only in MS did its TAV exceed 1, indicating a distinctive sourness profile for this sample. Succinic acid revealed TAVs < 0.03 in all the samples and thus contributed negligibly to sourness. Collectively, the TAV patterns reflect diverse organic acid metabolism among samples and underscore the heterogeneity of flavor formation mechanisms across fermentation systems.

### 3.3. Flavors Comparison of Spontaneously Fermented Industrial Sichuan Paocai from Different Producing Areas

**Figure 2:**
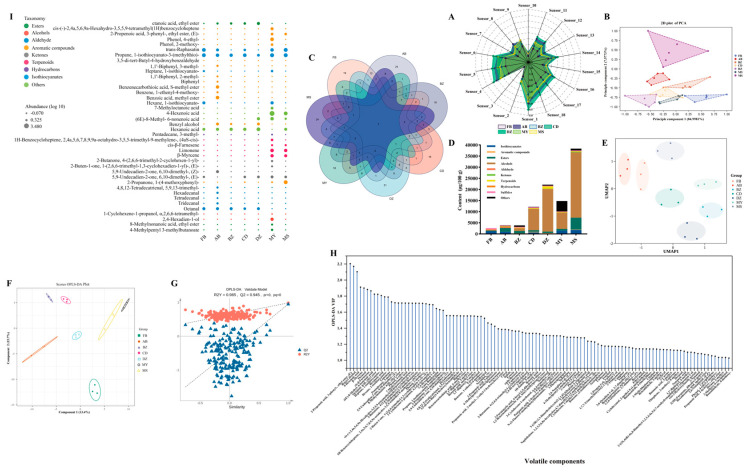
Radar plots (**A**) and principal component analysis (PCA) loadings plot (**B**) of the *E*-nose sensor response of different samples; Veen profile of volatile organic compounds (**C**); concentration of the different types of volatile organic compounds (**D**); UMPA analysis of volatile organic compounds (**E**); orthogonal partial least squares discriminant analysis (OPLS-DA) score plot (**F**), permutation test under 200 times for OPLS-DA (**G**), and variable importance (VIP) in the project score (**H**); heatmap of the VIP volatile organic compounds (VIP > 1.5, *p* < 0.05) content (**I**), the circle size represents the concentration of volatile organic compounds. FB represented Paocai fermented by fresh brine; AB represented Paocai fermented by aged brine; BZ, CD, DZ, MY, and MS represented industrial Paocai produced using traditional processes in Bazhong City, Chengdu area, Dazhou City, Mianyang City, and Meishan City, respectively.

E-nose was used to obtain an initial overview of the aroma profiles of industrial Sichuan Paocai produced in different regions (Figure 2A,B). The radar plots (Figure 2A) showed qualitatively similar response patterns across the 18 sensors, but with marked differences in the overall signal intensity. The radar-plot polygon area was largest for CD, with particularly strong responses at Sensor 1, Sensor 4, and Sensor 5 (sulfur-sensitive; Table 1), suggesting a higher abundance of sulfur-containing volatiles. By contrast, AB exhibited the lowest overall response, suggesting that its flavor intensity may be relatively weak. Principal component analysis (PCA) scores (Figure 2B) revealed a clear separation among samples, corroborating the differences in aroma profiles. To elucidate the chemical basis of these differences, volatile compositions were then characterized by comprehensive two-dimensional gas chromatography–mass spectrometry (Figure 2C–I).

A total of 294 compounds were detected by GC × GC–MS and classified into nine chemical groups: isothiocyanates, aromatics, esters, alcohols, aldehydes, ketones, terpenes, hydrocarbons, sulfides, and others (substance classification according to the order of the above categories). The number of compounds per sample was as follows: FB, 85; AB, 94; BZ, 89; CD, 89; DZ, 93; MY, 96; and MS, 91 (shown in supplementary materials). Only seven compounds were shared by all seven samples (including propane, 1-isothiocyanato-3-(methylthio)-, benzeneacetaldehyde, phenylethyl alcohol, anethole, tetradecane, dimethyl trisulfide, and pentanenitrile, 5-(methylthio)-), whereas each sample contained at least sixteen unique compounds (Figure 2C). The total volatile abundance (sum concentration) was lowest in FB and AB (Figure 2D), consistent with the E-nose results, whereas MS and DZ exhibited the highest totals, driven mainly by alcohols. Alcohols dominated the volatile profiles of industrial Paocai; in MS, total alcohols reached 29,375 μg/100 g. Prior work has shown that adding a small amount of liquor before fermentation can inhibit the growth of undesirable microorganisms [18]; some industrial producers may leverage this practice to stabilize fermentation and, indirectly, enhance volatile formation. By contrast, total alcohols in the household-style FB and AB samples were 66.008 and 263.319 μg/100 g, respectively. Samples with higher alcohol levels typically demonstrated a stronger overall aroma and fruit-like notes and also provided precursors for ester synthesis. Given the central role of esters in flavor, we further compared their levels and putative origins across samples. Esters were abundant overall, with MS (5302.495 μg/100 g) significantly exceeding the other groups, followed by AB (1633.861 μg/100 g) and BZ (1017.263 μg/100 g). Differences in ester levels likely reflect both the supply of alcohol precursors and the esterification enzyme activity. For instance, MS showed high alcohols and esters (adequate substrates and efficient esterification), whereas DZ had very high alcohols (19,023.233 μg/100 g) but comparatively low esters (591.422 μg/100 g), consistent with the limited esterification or diversion of substrates to competing pathways. Isothiocyanates—typical of cruciferous vegetables—exceeded 1000 μg/100 g in MS, MY, and FB, imparting pungent, mustard-like notes [28]. Beyond the total abundance, we also compared the class-level composition between household and industrial Paocai. Household Paocai differed from industrial products not only in total volatile levels but also in chemical composition. FB contained lower alcohols and aromatics than the industrial samples and demonstrated no detectable terpenes, whereas aldehydes, hydrocarbons, and sulfides were relatively higher. The absence of terpenes indicates a lack of floral and fruity nuances [29]. AB generally exhibited higher levels of aromatics, esters, alcohols, aldehydes, ketones, and terpenes than FB; this may also imply a superior flavor for AB, because perceived aroma arises from multi-compound interactions and the excessive intensity of individual compounds can generate a negative flavor. These patterns are closely linked to processing conditions, brine recycling frequency, and the resident microbial community.

Overall, our results suggest that industrial Paocai produced using traditional practices achieves a higher overall volatile aroma quality than household aged brine Paocai, characterized by greater total volatiles, higher alcohols and esters, and broader chemical diversity. This advantage likely derives from tighter process control (raw material ratio, salinity, temperature, and fermentation time), greater stability of the flavor-forming microbiota (particularly lactic acid bacteria), and the judicious use of auxiliary ingredients. In contrast, household aged brine fermentations are more susceptible to environmental fluctuations and brine recycling effects, which can destabilize the community and skew the alcohol–ester balance or promote off-notes, thereby reducing flavor harmony. For industrial production, maintaining batch-to-batch consistency while balancing aroma with taste attributes (e.g., texture, acidity, and salinity) remains essential for maximizing consumer acceptance.

### 3.4. The Differences in Flavors of Spontaneously Fermented Industrial Sichuan Paocai from Different Producing Areas

Uniform manifold approximation and projection (UMAP), a nonlinear, topology-informed dimensionality-reduction method that preserves both the local and whole structure, is widely used to reduce the dimensionality of high-dimensional complex data to low-dimensional space. This method can cluster samples with similar features together, and ensure that the reduced data still reflect the distribution characteristics and internal relations of the original data in low-dimensional space [30]. UMAP was used to visualize between-sample differences in volatile profiles, which revealed a clear sample distribution in low-dimensional space (Figure 2E). We next applied the orthogonal partial least squares discriminant analysis (OPLS-DA) to distinguish specific differential volatiles across groups. The OPLS-DA score plot (Figure 2F) mirrored the separations observed using UMAP and using the E-nose PCA, indicating consistent group discrimination across methods. A 200-iteration permutation test supported adequate model fit and predictive ability (Figure 2G). A total of 111 volatiles were identified as discriminant compounds (VIP > 1, *p* < 0.05; Figure 2H). Among them, 2-propenoic acid, 3-phenyl-, ethyl ester, (E)-, benzyl alcohol, and 4-hexenoic acid showed the highest variable importance (VIP > 2) and were major drivers of group separation. 2-Propenoic acid, 3-phenyl-, ethyl ester, (E)- occurred predominantly in MS and MY, whereas benzyl alcohol was ubiquitous and is associated with sweet, strongly fruity notes [31]. A heatmap of the top 40 features (VIP > 1.5) (Figure 2I) showed pronounced enrichment of 13 aromatic compounds in AB and MY. Although many of these volatiles are formed during fermentation, ingredient-borne aromatics also contribute substantially [32]. Representative compounds (mean ± SD, μg/100 g) included: FB—propane, 1-isothiocyanato-3-(methylthio)-(116.979 ± 5.419), and trans-raphasatin (101.356 ± 1.965); AB—benzoic acid, methyl ester (68.854 ± 27.187), and propane, 1-isothiocyanato-3-(methylthio)-(53.841 ± 19.523); BZ—propane, 1-isothiocyanato-3-(methylthio)-(38.906 ± 6.634) and hexanoic acid (34.018 ± 2.481); CD—propane, 1-isothiocyanato-3-(methylthio)-(78.584 ± 11.628); DZ—hexanoic acid (115.605 ± 13.794); MY—4-hexenoic acid (3037.967 ± 1127.446) and propane, 1-isothiocyanato-3-(methylthio)-(192.451 ± 17.458); MS—2-propanone, 1-(4-methoxyphenyl)-(220.344 ± 9.931), and 4-hexenoic acid (189.909 ± 79.862). Glucosinolate-derived isothiocyanates are widely recognized for their health-promoting effects. The frequent detection of propane, 1-isothiocyanato-3-(methylthio) across samples likely reflects glucosinolate degradation under acidic conditions [33]. Low-abundance terpenoids, such as β-Myrcene, cis-β-Farnesene, and limonene, were also detected in MY and MS; these are usually biosynthesized via the isoprenoid precursors isopentenyl diphosphate (IPP) and dimethylallyl diphosphate (DMAPP) [29]. Octanal was ubiquitous among samples and is described sensorially as fatty, soapy, lemon-like, and green [34]. Overall, the 111 discriminant volatiles collectively underpin the observed differences in Paocai flavor quality across regions and production systems.

### 3.5. Microbial Diversity and Community Structure in Spontaneously Fermented Industrial Sichuan Paocai from Different Producing Areas

**Figure 3:**
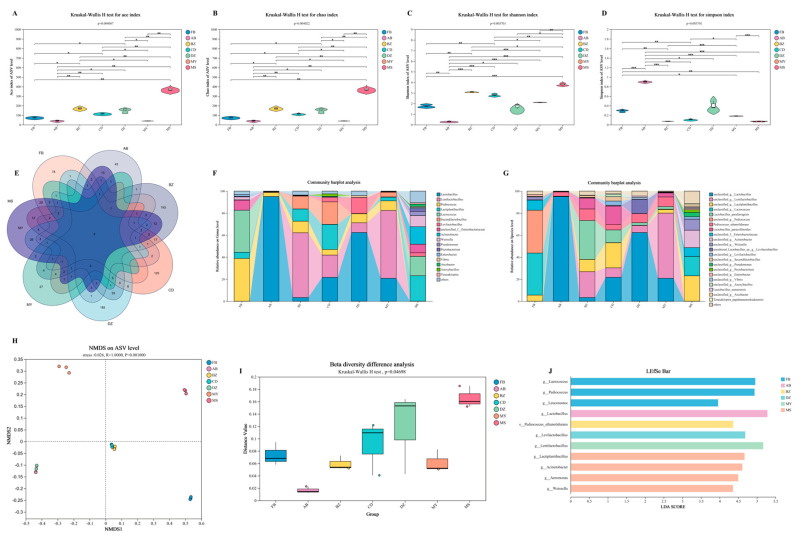
Kruskal–Wallis H test for the ACE index, Chao index, Shannon index, and Simpson index (**A**–**D**), respectively. 0.01 < *p* ≤ 0.05 marked *, 0.001 < *p* ≤ 0.01 marked **, *p* ≤ 0.001 marked ***; Veen profile of ASV (**E**); relative abundance of bacteria communities at genus and species levels in spontaneously fermented industrial Sichuan Paocai from different producing areas (**F**,**G**); NMDS analysis on the ASV level (**H**); Kruskal–Wallis H test-based beta diversity difference analysis (**I**); LEfSe analysis of bacteria communities in spontaneously fermented industrial Sichuan Paocai from different producing areas with an LDA log score threshold ≥ 3.5 (**J**). FB represented Paocai fermented by fresh brine; AB represented Paocai fermented by aged brine; BZ, CD, DZ, MY, and MS represented industrial Paocai produced using traditional processes in Bazhong City, Chengdu area, Dazhou City, Mianyang City, and Meishan City, respectively.

The diversity of microbial communities plays a critical role in sustaining fermentation system functions and shaping the complexity of product flavors. In this study, alpha diversity indices were compared across different samples (Figure 3A–D). Among them, the ACE and Chao indices reflected community species richness, while the Shannon and Simpson indices measured community diversity. In general, higher ACE and Chao indices correspond to greater species richness, whereas a higher Shannon index indicates greater community diversity, with the Simpson index showing the opposite trend [35]. The results demonstrated that MS samples exhibited the highest ACE, Chao, and Shannon indices, suggesting the richest species composition, with 728 ASVs, 204 species, and 171 genera detected. In contrast, AB and MY samples consistently displayed the lowest richness indices. Wilcoxon rank-sum tests further confirmed significant differences in the microbial diversity indices among Paocai samples from different production regions (*p* < 0.05). A Venn plot (Figure 3E) provided additional visualization of microbial composition, revealing that only two ASVs, g__*Lactobacillus* and g__*Lactiplantibacillus*, were shared across all samples, while most other microorganisms exhibited strong region-specific distributions.

At the genus level, distinct microbial profiles were observed. FB samples were dominated by *Pediococcus* (38.9%) and *Lactococcus* (38.2%), whereas AB and DZ were mainly composed of *Lactobacillus*. BZ and MY were characterized by *Lentilactobacillus*, while CD harbored a mixed composition, including *Lactobacillus*, *Lentilactobacillus*, *Lactiplantibacillus*, and *Secundilactobacillus*. In contrast, MS showed the greatest microbial diversity, dominated by *Acinetobacter*, *Lactococcus*, *Weissella*, and *Lactiplantibacillus*. Nevertheless, species-level identification remains constrained by current methodological limitations [36]. Among highly abundant microorganisms, only *Lactobacillus*_*parafarraginis*, *Lactobacillus*_*paracollinoides*, *Pediococcus*_*ethanolidurans*, and *Lactobacillus*_*namurensis* were successfully identified, and these species were consistently involved in Paocai fermentation across diverse origins [6].

Multivariate statistical analyses provided additional insights into differences in microbial composition. Non-metric multidimensional scaling (NMDS), based on Bray–Curtis distances, effectively reduced high-dimensional data into a lower-dimensional space, thereby enabling clear visualization of the sample differences. The NMDS analysis yielded a stress value of 0.026, indicating excellent model interpretability. Moreover, the NMDS distribution revealed that DZ and AB shared relatively similar microbial compositions, as did BZ and CD. The beta diversity analysis further confirmed intergroup differences, with statistical testing indicating significant variation among samples (*p* = 0.04698), highlighting marked regional differentiation of Paocai microbial communities. Subsequently, the LEfSe analysis was performed to identify the taxa responsible for these distinctions. The LDA discriminant analysis at both the genus and species levels revealed ten genera and one species with significantly different relative abundances across the seven production regions (LDA > 3.5, *p* < 0.05). Specifically, FB was enriched in g__*Pediococcus*, g__*Lactococcus*, and g__*Leuconostoc*; AB in g__*Lactobacillus*; BZ in s__*Pediococcus ethanolidurans*; DZ in g__*Levilactobacillus*; MY in g__*Lentilactobacillus*; and MS in g__*Lactiplantibacillus*, g__*Acinetobacter*, g__*Aeromonas*, and g__*Weissella*. Collectively, these taxa represent key microbial markers distinguishing Sichuan Paocai from different geographical regions.

### 3.6. Multivariate Analysis of Microorganisms and Flavor Compounds

**Figure 4:**
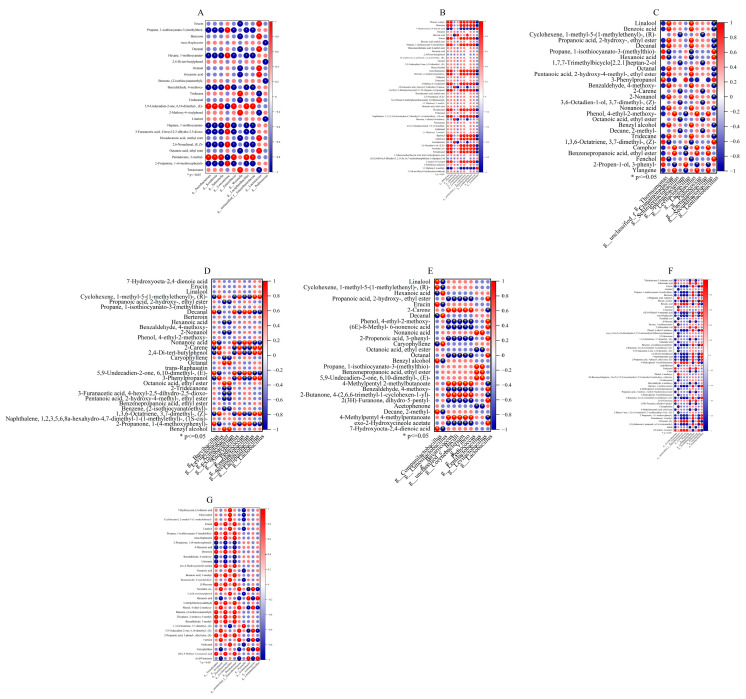
The Spearman’s correlation between VIP volatile organic compounds (VIP > 1, *p* < 0.05) and the top 10 genus level (relative abundance) microorganisms in each group. (**A**–**G**) represent FB, AB, BZ, CD, DZ, MY, and MS, respectively. FB represented Paocai fermented by fresh brine; AB represented Paocai fermented by aged brine; BZ, CD, DZ, MY and MS represented industrial Paocai produced using traditional processes in Bazhong City, Chengdu area, Dazhou City, Mianyang City, and Meishan City, respectively. The “*” in the circles represent that the difference between them was significant at the *p* < 0.05 level.

Microbial metabolites represent a major source of flavor compounds [37]. To elucidate the intrinsic associations between microorganisms and flavor formation in Paocai fermentation systems, we employed Spearman’s correlation analysis to assess the relationships between the top 10 microbial genera (by relative abundance) and key volatile flavor compounds (VIP > 1) across samples from different origins (Figure 4A–G). The results were visualized in a heatmap (Figure 4). Overall, both positive and negative associations emerged between microbial taxa and flavor compounds, underscoring the pivotal role of microorganisms in driving the generation and transformation of flavors.

Within the lactic acid bacteria (LAB) branch, several genera were identified as core functional groups responsible for producing fresh and pleasant flavor notes. In the FB samples, g__*Lactiplantibacillus*, g__*Lactococcus*, g__*Pediococcus*, and g__*Weissella* exhibited significant positive correlations (*p* ≤ 0.05) with multiple desirable compounds. These included Decanal, which imparts a refreshing citrus aroma [38], whose accumulation strongly paralleled the abundance of these LAB. Similarly, Linalool, a representative floral aroma compound, was closely linked to g__*Lactiplantibacillus* and g__*Lactococcus*. Furthermore, 2,6-nonadienal (E, Z), which is responsible for a cucumber-like freshness [39], showed significant positive correlations with g__*Leuconostoc*. Additionally, g__*Lactococcus* was associated with ester formation, such as Octanoic acid ethyl ester, a compound that provides sweet, fruity notes and markedly enhances the overall flavor harmony. In AB samples, g__*Weissella* and g__*Leuconostoc* were likewise correlated with aldehydes (e.g., Undecanal) and esters (e.g., Octanoic acid ethyl ester), a trend also evident in industrial fermented products. Collectively, these results emphasize that promoting LAB growth is fundamental to shaping the fruity, floral, and fragrant flavor profile of Paocai.

Despite the typically low pH, high acidity, and elevated salinity of Paocai fermentation systems, certain opportunistic or undesirable taxa (e.g., *Enterobacter*) were able to persist. Notably, a high abundance of such bacteria was detected exclusively in family-scale fermentations. These taxa generally displayed negative correlations with isothiocyanates, suggesting that these bioactive compounds may restrict their proliferation through antimicrobial activity. Conversely, in systems with insufficient inhibitory metabolites, opportunistic bacteria more readily overcame ecological barriers and occupied niches. Previous studies have demonstrated that isothiocyanates, derived from glucosinolate degradation, effectively suppress Gram-negative bacteria by disrupting the membrane integrity and perturbing metabolic pathways [40]. Thus, they likely represent an important natural selective pressure in Paocai ecosystems. Our findings further suggest that optimizing raw materials or processing conditions to stimulate the accumulation of active metabolites, such as isothiocyanates, could offer a practical strategy to enhance the microbial safety and ecological stability of home-scale Paocai fermentation.

## 4. Conclusions

This study highlights the strong regional heterogeneity of Sichuan Paocai in both microbial composition and flavor formation. Lactic acid bacteria were identified as the core functional group responsible for acid production and the generation of key volatile compounds that shape the sensory characteristics of Paocai. Distinct microbial markers differentiated Paocai from different regions, reflecting the impact of local fermentation environments on microbial ecology. Moreover, the negative association between miscellaneous bacteria and isothiocyanates suggests that these bioactive compounds act as natural barriers, enhancing the system stability and safety. Overall, industrial Sichuan Paocai fermented using traditional processes may exhibit superior flavor compared with family-scale spontaneous fermentation, largely due to the use of diverse auxiliary ingredients and standardized raw material ratios. Moreover, the precise control of salinity, temperature, and fermentation time establishes a more stable physicochemical environment, thereby facilitating the rapid colonization and metabolic activity of dominant lactic acid bacteria. In contrast, family-scale spontaneous fermentation systems often display greater randomness in microbial community composition and are more vulnerable to exogenous microorganisms or environmental disturbances, leading to variability in flavor formation. Collectively, these findings suggest that standardization and regional characteristics jointly shape the flavor of Sichuan Paocai: the former ensures product stability and safety, while the latter imparts distinctive regional traits, providing a scientific foundation for future industrial optimization and regional brand development.

## Figures and Tables

**Table 1:** Electronic nose sensor array characteristics.

Sensor Name	Performance Description	Sensor Name	Performance Description
S1	propane and fumes	S10	hydrogen
S2	alcohol, fumes, isobutane, and formaldehyde	S11	liquefied gas and alkanes
S3	ozone	S12	liquefied gas and methane
S4	hydrogen sulfide	S13	methane
S5	ammonia	S14	flammable gas and fumes
S6	toluene, acetone, ethanol, and hydrogen	S15	fumes and isobutane
S7	methane, natural gas, and biogas	S16	sulfides
S8	liquefied gas	S17	nitrides
S9	toluene, formaldehyde, benzene, alcohol, and acetone	S18	acetone and alcohol

**Table 2:** The taste activity value of organic acids in spontaneously fermented industrial Sichuan Paocai from different producing areas.

Compounds	Taste Attributes	Threshold ^a^ (mg/g)	TAV ^b^						
			FB	AB	BZ	CD	DZ	MY	MS
Lactic acid	Sour	1.261	4.262 ± 0.036	6.896 ± 0.162	13.238 ± 0.35	14.988 ± 0.034	11.565 ± 0.06	13.581 ± 0.133	4.296 ± 0.127
Oxalic acid	Sour	0.504	17.228 ± 0.017	24.894 ± 0.363	6.357 ± 0.056	14.888 ± 0.052	0.221 ± 0.001	16.628 ± 0.198	23.411 ± 0.658
Citric acid	Sour	0.499	0.066 ± 0	0.104 ± 0.003	0.079 ± 0.005	0.3 ± 0.011	0.054 ± 0.001	0.059 ± 0.001	1.561 ± 0.062
Malic acid	Sour	0.496	7.357 ± 0.176	8.927 ± 1.478	2.724 ± 0.2	3.177 ± 0.685	1.526 ± 0.044	5.286 ± 0.072	13.294 ± 0.372
Succinic acid	Sour	0.106	0.001 ± 0	0.001 ± 0	0.001 ± 0	0.002 ± 0	0.001 ± 0	0.002 ± 0	0.003 ± 0

^a^ The threshold of each compound (in water) was obtained from Rotzoll, Dunkel and Hofmann [26]. ^b^ TAV, taste active value.

## Data Availability

The original contributions presented in the study are included in the article/Supplementary Material, further inquiries can be directed to the corresponding author.

## Supplementary Materials

The following supporting information can be downloaded at: https://www.mdpi.com/article/10.3390/foods14183232/s1, Flavor components of different samples.
